# Editorial: Mindfulness and Eating Behavior

**DOI:** 10.3389/fpsyg.2018.01986

**Published:** 2018-10-12

**Authors:** Michail Mantzios

**Affiliations:** Department of Psychology, Birmingham City University, Birmingham, United Kingdom

**Keywords:** mindfulness, mindful eating, obesity, eating behavior, self-regulation

Research associating mindfulness and eating behavior has a short history that starts with the first publication almost two decades ago (Kristeller and Hallett, 1999). Since then, a consistent body of research developed our understanding for the treatment of eating disorders (e.g., Kristeller and Wolever, 2010) and obesity (e.g., Mantzios and Wilson, 2015), and a plethora of methods achieving the end goal of self- and weight- regulation have been proposed and investigated (e.g., Tapper, 2017). From brief mindful eating interventions (Allirot et al., 2018) and mindful diaries (Hussein et al., 2017) to conventional mindfulness programmes (Raja-Khan et al., 2017), the field has been expanding and pilot and feasibility studies have become larger randomized clinical trials (e.g., Loucks et al., 2015; Mason et al., 2016) and population studies (Camilleri et al., 2015). Existing research has been useful in advancing understanding and practices around mindfulness, but in many ways, it appears as if we are taking a more informed and evidence-based path, which indeed, is depicted in this Research Topic of “mindfulness and eating behaviors.”

## Overview of contributions

The research topic opens with a manuscript by Tapper and Ahmed who present a controlled experiment around decentering, and investigate the mechanism of cognitively accessible goals to explain resistance to chocolate. When decentering was compared to a control group in assessing cognitive accessibility through the “word stem completion task,” researchers found a significantly greater number of words relevant to health and weight loss in the decentering condition. The second manuscript by Masuda et al. reports a cross-sectional study investigating mindfulness as a moderating factor between eating disorder related cognitions and eating disorder behaviors across different ethnical backgrounds. While the moderation was true for the White American sample, mindfulness failed to moderate this relation between cognitions and behaviors for Asian and Black Americans. Findings suggest individual differences may influence the effectiveness of mindfulness practices, and propose future research with a greater emphasis on minority groups, as well as greater explorations of cross-cultural differences. The third manuscript (Soler et al.) compared findings across five groups (including four different clinical samples and a control group) on the experience of eating through a unique method of “direct experiential comprehension” of eating. Among the findings, importantly, healthy control and obesity patients scored higher on the “direct experience index” than the anorexia and bulimia samples. The findings suggest a close association between healthy control and obesity patients, meaning that there may be further considerations when we recommend elements surrounding mindful eating to obesity patients, or explorations of healthy populations enabling pilots that may be applicable to obesity samples. In the fourth manuscript, Brewer et al. put forward mechanisms for newer interventions that are relevant to habit formation, operant conditioning and intrinsic rewards. Discussions around intrinsic rewards have been long overdue in the field of mindfulness and eating, and may offer a potential variable in long-term adherence to both mindfulness and weight-regulation. Although personally I feel more inclined toward an approach that goes along with Brewer et al.'s model presented in this issue (e.g., see Mantzios and Giannou, 2018), the added elements of traditional contemplative practices (such as cultivating spiritual engagement) may propose a more in-depth practice. In the fifth manuscript, Kristeller and Jordan do indeed tap into the additional value of spirituality within the mindfulness-based eating awareness training programme, and explore meaningful spiritual engagement as a benefit to the enhancement of self-regulatory processes. For novice participants and/or patients, it may be easier to start with secular practices (as seen in Soler et al.; Tapper and Ahmed) and build on them to create more sustainable practices (as in Brewer et al.). However, they may eventually transition onto non-secular practices, which may be deeper, and most likely, more effective and rewarding interventions, and this is definitely, what Kristeller and Jordan propose through their findings. Last, Egan and Mantzios, through a qualitative exploration, investigate perceptions of self-kindness and self-compassion, and draw associations between self-kindness and self-indulgence, while emphasizing an overarching theme of caring equally for body and mind (see Mantzios and Egan, 2017), which reflects on mindfulness practices and the way we need to perceive the non-evaluative and non-judgmental elements of engagement with food. The descriptions of the manuscripts presented in this editorial are by no means inclusive of the full content, but more likely an invitation to read and comment on the methods and findings of the manuscripts further. Throughout the Research Topic, the authors engage in discussions and offer deeply insightful comments on the findings, as well as inspiring projections for future research.

Indeed, the collection of work proposes exceptionally well-executed experiments, in-depth qualitative explorations, examinations of the role of ethnical background and spirituality in mindfulness and mindfulness programmes, and different theoretical directions. Jointly, the manuscripts put forward stimulating directions for future cross-sectional, experimental and longitudinal research in mindfulness and eating behaviors. Looking forward and at the wider range of investigation, there may be a need for a consensus manuscript that puts forward standardized practices and methods that can be used easily and freely, to compare findings and replicate significant and influential research in the field. Together we may be able to make a difference, and together we may be able to change the impact of mindfulness on the quality, quantity and the ways that we eat.

## Author contributions

The author confirms being the sole contributor of this work and has approved it for publication.

### Conflict of interest statement

The author declares that the research was conducted in the absence of any commercial or financial relationships that could be construed as a potential conflict of interest.
